# Structure of the Scleroglucan Biopolymer in Aqueous Solutions of Inorganic Salts and Ionic Liquids

**DOI:** 10.1021/acsomega.5c02212

**Published:** 2025-06-11

**Authors:** Zsófia Vargáné Árok, Gábor Dávid Vass, Andrej Jamnik, Matija Tomšič, Istvan Szilagyi

**Affiliations:** † MTA-SZTE Momentum Biocolloids Research Group, Department of Physical Chemistry and Materials Science, Interdisciplinary Research Center, 316425 University of Szeged, H-6720 Szeged, Hungary; ‡ Faculty of Chemistry and Chemical Technology, 37663 University of Ljubljana, Večna Pot 113, Ljubljana SI-1000, Slovenia

## Abstract

The effect of inorganic salts and ionic liquids (ILs) on the solution properties of scleroglucan (SG), an increasingly used and researched natural biopolymer, is reported here. Various rheological, electrophoretic, and small-angle X-ray scattering experiments were carried out to assess rheological and charge-related features, as well as the characteristic transitions in terms of the system’s viscoelastic and structural features were unambiguously determined. The SG solution shows pseudoplastic properties at moderate polymer concentrations. Multivalent metal ions tend to stabilize the strong elastic character of the pseudoplastic SG solutions even at elevated salt concentrations relevant to many SG applications due to the specific interactions with the polymer chains. The affinity of the metal ions to the SG chains followed the calcium­(II) > magnesium­(II) > sodium­(I) order, which was reflected in different charge values at the same salt concentrations. The coherent structure of the SG was interrupted at high monovalent salt concentrations and was recovered by adding imidazolium-based ILs (C2-mimCl and C4-mimCl) of different alkyl chain lengths. These results shed light on the importance of chemical additives to alter the pseudoplastic and viscoelastic features of the studied SG solutions, which makes such biopolymers promising candidates in applications where high-viscosity samples with distinct viscoelastic character are needed.

## Graphical abstract

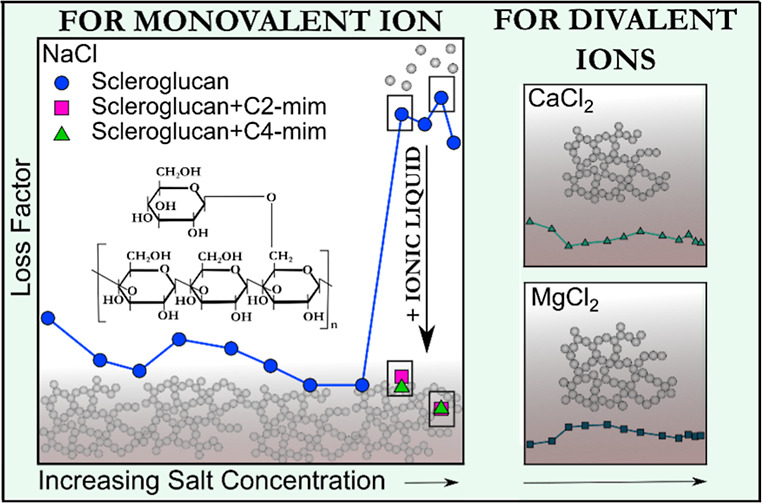

## 1 Introduction

Scleroglucan (SG) is the collective name for natural polysaccharides, which are produced by the genus Sclerotium fungi during fermentation processes,^1^ while the most important ones for large-scale production are the fungal species Sclerotium rolfsii and Sclerotium glucanicum.^2–4^ They produce such polymers of similar chemical structure with some deviations in branching frequency, in length of the side chains, and thus, in molecular weight.^5^ In general, the repeating unit of SG consists of four β-d-glucopyranose units, three of which are connected to each other by 1,3 bonds in a chain, while the fourth unit forms the side chain through a 1,6 bond (Scheme 1).^6–8^ Moreover, SG was found to be biodegradable and nontoxic.^9^

**Scheme 1:**
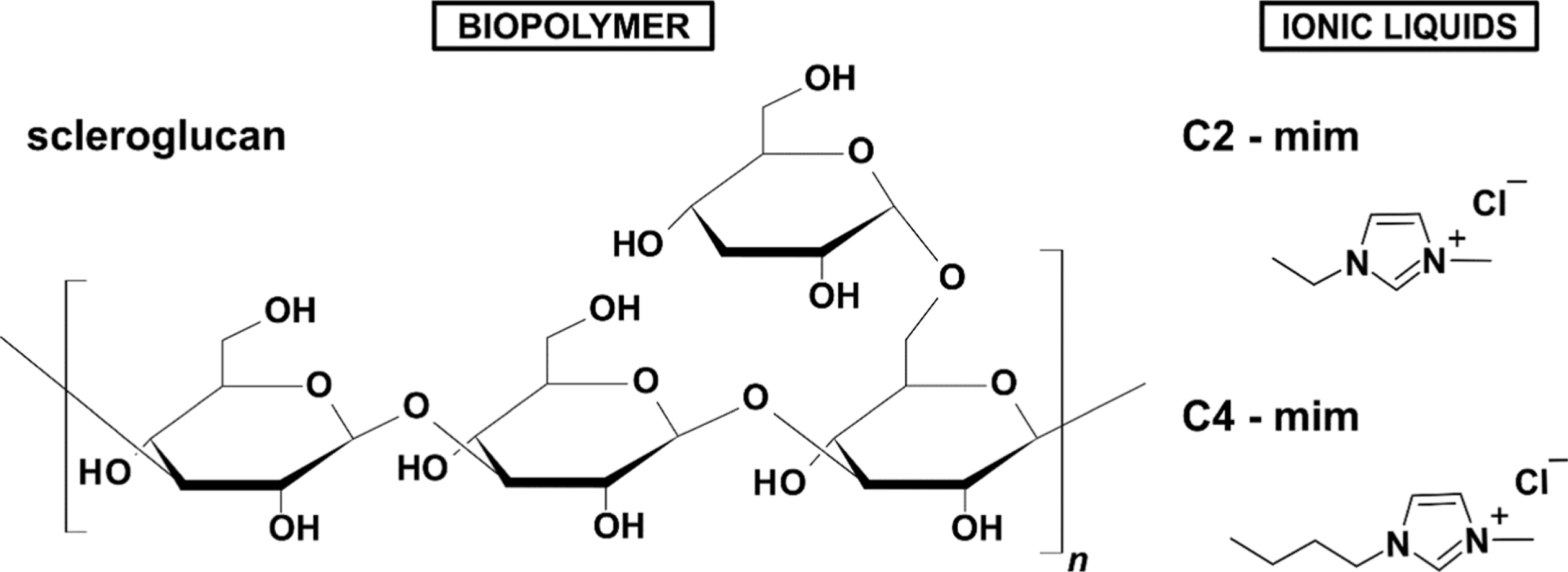
Chemical Structure of the SG and the ILs Used^a^

a C2-mimCl: 1-ethyl-3-methylimidazolium chloride, C4-mimCl: 1-butyl-3-methylimidazolium chloride.

Applications of SG include the areas of construction engineering, additives in foods, cosmetics and printing inks, thickener and stabilizer in paints, adhesives, and pesticides.^2,10–13^ For instance, no specific interactions were observed between SG and salivary proteins, which is an important fact toward SG application in food products and related manufacturing procedures.^14^ Besides, major research efforts were made to explore its applicability in enhanced oil recovery processes.^15,16^ Its effectiveness may exceed that of many other macromolecules used in polymer flooding^17–20^ because the viscosity of SG solutions is less affected by the salinity and pH due to its less ionic character.^21^ For example, viscosity of SG remained unchanged for months at elevated temperature and high salinity compared to partially hydrolyzed polyacrylamides,^22^ and its efficiency in sweeping of oil residual was also confirmed in carbonate reservoirs even at relatively lower polymer concentrations.^23^ Furthermore, surfactants can induce significant increase in the viscosity of SG solutions via entrapment into the chain and/or advantageous conformational changes upon surfactant–polymer assemblies.^15^ Moreover, interaction of SG with surfaces can be also tuned by adding hydrophobic moieties to the hydrophilic chains,^24^ while the presence of nanoparticles and subsequent formation of extensive particle-SG network led to improved thickening behavior.^16^

Concerning rheological properties governing the viscosity profile of SG solutions, they are important parameters in the above applications. Rotational and oscillatory rheological studies revealed that SG may form coherent gel-like structure at appropriately high concentration or macromolecular solutions in more diluted aqueous solutions.^25^ Time-dependent rheology results indicated a significant delay to remove shear history, and the coherent form was characterized as a weak gel system. The effect of pH on such a sol–gel transition was investigated in rheological experiments, in which the acidity was tuned in situ by inducing ester hydrolysis and subsequent formation of carboxylic acids.^26^ It was reported that the transition of individual SG chains in solution to their typical triple-helical state can be enhanced by the released acids.

The latter example indicates that the rheological features and sol–gel transition conditions can be tuned by chemical additives. This was also demonstrated in polymer solutions used for oil recovery.^27^ Such additives include nanoparticles,^27,28^ surfactants,^28–30^ and ionic liquids (ILs).^31–33^ The latter were found to be particularly useful in oil reservoirs under harsh conditions due to their enhanced thermal and chemical stability. In general, ILs are low melting point substances usually composed of organic cations and inorganic anions forming various bulk structures, and many of them are liquid at room temperature.^34^ Advantageous properties involve their negligible vapor pressure, high stability, and tunable structure.^35^ Use of ILs is widespread and booming in areas such as electrochemistry,^36^ materials science,^37–39^ analytical chemistry,^40^ pharmaceutical,^41^ and food industry,^42^ as well as in enhanced oil recovery in both production^43^ and post-treatment.^44^ Poly-ILs were also used to prepare cellulose-based hydrogels for strain sensor application.^45^ Based on this information, the growing number of IL applications can be foreseen in areas where polymers are used, and their solution form has to be adjusted by appropriate additives. Despite the fact that ILs are promising candidates to adjust SG sol–gel solution features, no comprehensive studies have been performed yet in SG-IL systems.

Therefore, the present work reports on the rheology and charge features of SG solutions in the presence of salts of different valences and concentrations as well as of ILs. The viscoelastic features were assessed in rotational and oscillatory modes and the structural characteristics by small-angle X-ray scattering (SAXS) experiments, while electrophoretic light scattering measurements were performed to determine the charge properties in aqueous SG solutions under various experimental conditions. Based on the results, quantitative information is given on the effect of solution composition on tuning the rheological and structural nature of the SG systems studied.

## 2 Experimental Methods

### 2.1 Materials

The SG polymer was kindly offered by Cargill (product no. Actigum CS6) and used as received. In general, these polymers have average molecular masses ranging from 1 × 10^5^ to 6 × 10^6^ Da.^46^ The salt concentration was adjusted by NaCl, CaCl_2_, and MgCl_2_ (all from VWR in solid form). The 1-ethyl-3-methylimidazolium chloride (C2-mimCl, ≥ 98%) IL was purchased from IO-LI-TEC, while 1-butyl-3-methylimidazolium chloride (C4-mimCl, ≥ 98%) was obtained from Sigma-Aldrich. The salts and ILs were used without further purification. Ultrapure water was produced with an ADRONA B30 device. The measurements were carried out at 25 °C.

### 2.2 Preparation of Polymer Samples

Calculated amount of SG powder was dissolved in water or in salt solutions in 5 g/L concentration followed by stirring for 16 h. Dilution series were obtained from the most concentrated salt-polymer solution by dilution with the polymer solutions at the respective concentrations without added salt. The final electrolyte concentrations were between 0.2 and 200 g/L.

### 2.3 Rheology

An Anton Paar MCR 302 modular rheometer was used for rotational and oscillatory measurements. The employed geometry was a DG26.7-SS double-gap measuring system in both cases. For rotational rheology, 1–1000 1/s shear rates were applied. Given the application of very high shear rates up to 1000 1/s, it is crucial to emphasize that the data collected were rigorously validated to ensure reliability, with careful measures taken to prevent edge fracture and slippage during the experimental process. Oscillatory measurements were performed with an amplitude sweep technique at a constant frequency of 10 Hz.

### 2.4 Electrophoresis

To assess charge properties of the SG in solutions, electrophoretic light scattering measurements were carried out with a Litesizer 500 device using a Univette accessory (Anton Paar). The reported electrophoretic mobility data were the mean of five individual measurements.

### 2.5 Small-Angle X-ray Scattering

The SAXS measurements were carried out using an in-lab-modified Kratky-type camera (Anton Paar KG, Graz, Austria) with a focusing multilayer optics (Göbel mirror) and a block-collimation unit. It was connected to a conventional X-ray generator (GE Inspection Technologies, Seifert Isodebyeflex 3003; Cu-anode operating at 40 kV and 50 mA; λ (Cu *K*_α_) = 1.54 Å). The samples were put into a cylindrical quartz capillary (cross-section diameter of 1 mm and wall thickness of 10 μm), thermostated to 25 °C, and measured for 1 h. SAXS intensities were recorded using the Mythen 1 K detector (Dectris, Switzerland) in the range of the scattering vector (*q* = (4π/λ)­sin­(θ/2), where θ is the scattering angle) from 0.08 to 7 nm^–1^. The resulting scattering data were corrected for background and solvent scattering and brought to an absolute scale,^47^ and they were still experimentally smeared due to the finite dimensions of the primary beam.^48^

### 2.6 Fitting the SAXS Data

To fit the SAXS data, the so-called classical approach was followed, which is described in more detail elsewhere.^49,50^ Note that only a very brief description with the basic equations is given here and find some further details on the impact of the experimental smearing^51^ effect on the SAXS data fitting procedure in the Supporting Information.^49–51^ The SAXS data of the homogeneous polymer solutions can usually be described by an expression based on the Ornstein–Zernike function, which provides an expression for the scattering intensity in Lorentzian form as^52,53^

1 $$I\left(q\right)=\frac{C}{1+q^{2}\xi^{2}}$$

where *C* is a constant and ξ the dynamic correlation length, which corresponds to the distance to which the movement of the flexible polymer chains in the polymer solution is correlated. However, as the polymer concentration increases and the system further transitions to the gel state, the more persistent elastic polymer entanglements occur and result in the need for an additional correlation length parameter to accurately describe the corresponding SAXS data, namely, the static correlation length Ξ. In the low-*q* region of the SAXS scattering curve, additional excess scattering occurs, which can be treated with the help of the Debye–Bueche formalism^54,55^ (squared Lorentzian) leading to the following scattering intensity expression^53,56,57^

2 $$I\left(q\right)=\frac{A}{1+q^{2}\xi^{2}}+\frac{B}{{\left(1+q^{2}\Xi^{2}\right)}^{2}}$$

where *A* and *B* are constants. The static correlation length Ξ corresponds to the size of the polymer entanglements in the structure. These similar equations for the scattering intensity have been successfully tested on numerous small-angle neutron scattering and SAXS data of gels and solutions.^49,50,53,56–60^

## 3 Results and Discussion

### 3.1 Structural Coherency of SG in Water

First, the SG concentration at which sol–gel transition occurs was determined by rotational and oscillatory rheology measurements in pure aqueous SG solutions of concentrations in the range from 0.1 to 2.5 g/L. The results obtained by rotational rheology measurements are presented in Figure 1 (data also given in Table S1) in the form of the flow curves and the concentration dependencies of some rheological parameters. In the case of the flow curves, uncertainties were established prior to the measurements for the 1 g/L concentration SG solution, where the given relative standard deviation was below 5% in all cases.

**Figure 1:**
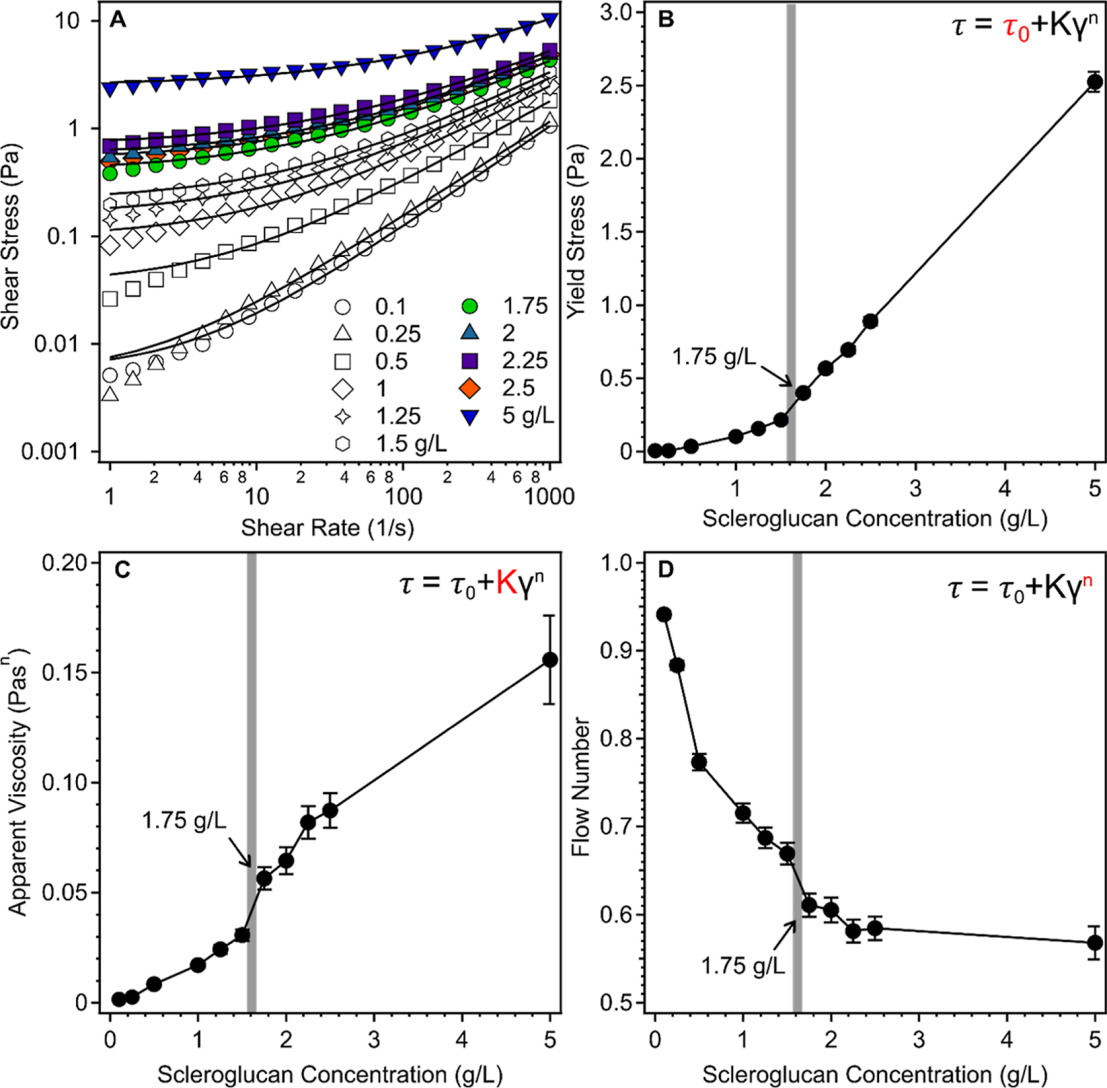
Flow properties of SG at different polymer concentrations. Flow curves (A) and parameters obtained by fitting them with the Herschel-Bulkley model (see inset). Yield stress (B), apparent viscosity (C), and flow number (D) as a function of the SG concentration. The sol–gel transition regime is indicated by gray areas in (B–D). The solid lines in (A) are the results of the fits with eq 3, while they are eye guides in (B–D).

The concentration-dependent flow curves (shear stress versus shear rate) of aqueous SG solutions (see Figure 1A) were fitted with the Herschel–Bulkley model (eq 3)^61^ to obtain the yield stress (Figure 1B), the apparent viscosity (Figure 1C), and the flow number values (Figure 1D)

3 $$\tau =\tau_{0}+K\gamma^{n}$$

where τ is the shear stress, and τ_0_, *K*, and *n* represent the yield point, the apparent viscosity, and the flow number, respectively.

These values depend on the gelation state of the polymer systems. Namely, the yield stress is equal to the minimum shear of the irreversible plastic deformation, and the apparent viscosity is related to the resistance of the polymer solution against the flow, while the flow number is close to unity for a macromolecular sol and smaller for gels of coherent structure. The state of the solutions can be deduced from the shape of the rheological flow curves (Figure 1A). The Newtonian liquids (sols) exhibit a linear course of the flow curves (constant viscosity), while with the appearance of pseudoplastic behavior of the system, the deviations from linearity appear (viscosity is not constant).^62^ Such a transition between these two system states can clearly be seen by increasing the SG concentration, i.e., the flow curves are linear at low and nonlinear at higher SG concentrations. The yield stress is zero at the lowest (0.1 g/L) SG dose, indicating the Newtonian character of this liquid sample (see Figure 1B). As the concentration of SG increases, an initial gentle increase is followed by a steeper one indicating a significant change in the coherency of the samples at higher SG concentrations.^18^ This was also confirmed by the 2.5 Pa yield stress at a 5 g/L concentration, which indicates the presence of strong coherent interactions between the biopolymers. Apparent viscosity values (Figure 1C) also increased rapidly with increasing SG concentration, while the relevant flow number values (see Figure 1D) gradually decreased from one, reflecting the predominance of intermolecular interactions and pseudoplastic nature of the system at higher SG concentration. The tendencies in the data obtained from the concentration-dependent flow curve fits indicate break points around the polymer concentration of 1.75 g/L, which designate some qualitative changes in interparticle interactions and possibly a sol–gel transition regime. To further investigate this phenomenon, oscillatory rheological measurements were performed, and the loss factor values were determined in the same SG concentration regime, as shown in Figure 2.

**Figure 2:**
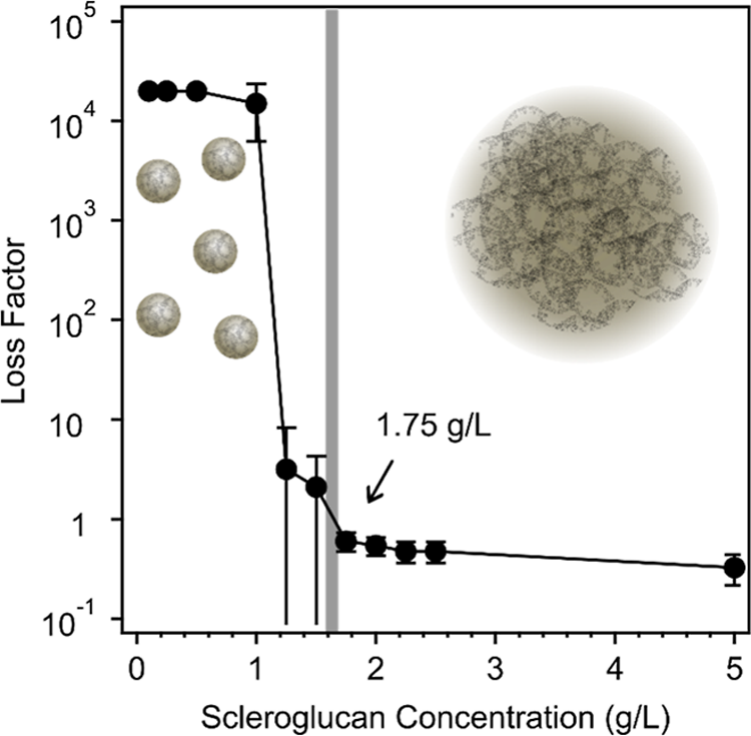
Loss factor data calculated for SG solutions at different polymer concentrations based on the measured storage and loss moduli values using eq 4. The corresponding storage and loss moduli data are presented in Table S2 in the Supporting Information.

This parameter, which reflects solution structure of polymers,^17,18^ is calculated as the ratio of the storage and loss moduli^63^

4 $$\tan\delta =G^{\prime \prime }/G^{\prime }$$

where tan δ is the loss factor, *G*″ is the loss modulus, and *G*′ represents storage modulus (the latter two are usually expressed in units of Pa). The storage modulus is related to the elastic properties of the material and provides information on the deformation energy stored by the elastic polymer structure, while the loss modulus reflects the magnitude of the dissipated deformation energy and the viscous behavior. Accordingly, their values are similar, i.e., the loss factor is close to unity, in the case of samples close to the sol–gel transition, or they can also differ significantly for gels and Newtonian liquids.^64^ The transition from sol to gel is reached as soon as the loss factor becomes less than one, while its value is ideally zero for solid-like elastic gels, which is not the case in our samples due to the moderate polymer concentration. A significant steep drop in the loss factor value can be observed in Figure 2 at SG concentrations just above 1 g/L and settles at values lower than that above around 1.50 g/L of SG in the sample.

In the next step, the structural properties of the aqueous SG system were investigated using the SAXS method and fitting the corresponding SAXS data by eqs S3 and S4. The former was derived from eq 1, which is normally used for liquid polymer solutions, and the latter from eq 2, which is normally used for polymer gels, as briefly explained in the online Supporting Information. The resulting SAXS data with the corresponding fits are shown in Figure 3, and the resulting fit parameter values are given in Table 1.

**Figure 3:**
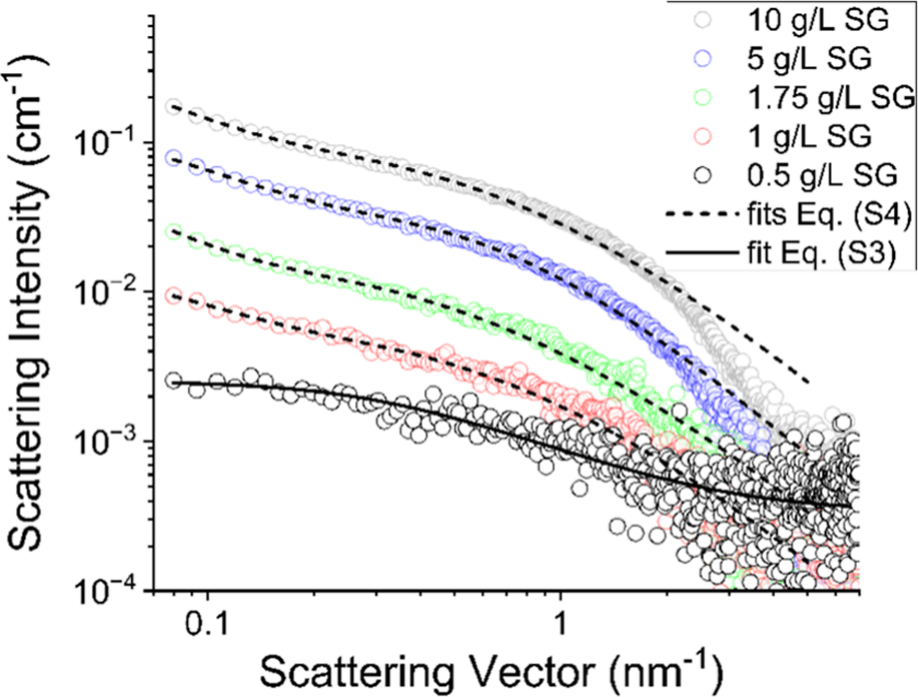
Experimental SAXS curves of pure aqueous samples with different concentrations of SG (symbols) and the corresponding fits according to eq S3 for the 0.5 g/L sample (solid line) and according to eq S4 for the others (dashed lines). Error bars in the low *q* regime are within the size of the symbols.

**Table 1:** Resulting Fitting Parameters of SAXS Data for Aqueous Samples with Different Concentrations of SG: the Dynamic Correlation Length, ξ, and the Static Correlation Length, Ξ

*c*_SG_ (g/L)	ξ (nm)	Ξ (nm)
0.5	3.2 ± 0.2	
1	2.3 ± 0.1	10 ± 1
1.75	2.7 ± 0.1	17 ± 2
5	1.8 ± 0.1	10 ± 1
10	2.5 ± 0.1	14 ± 1

Interestingly, only the sample with the lowest SG concentration, i.e., 0.5 g/L, could be successfully fitted by eq S3 (solid line in Figure 3), indicating that only the structure in this sample could be adequately described by only one structural parameter, the dynamic correlation length ξ of about 3.2 nm. As can be seen in Figure S2 in the Supporting Information, the fits of eq S3 were poor for other investigated samples, suggesting the use of eq S4 in these cases. The resulting fits of the latter are shown in Figure 3 (dashed lines) and are much better, especially in the innermost region of the SAXS curves, at low values of the scattering vector *q*, where the information about larger dimensions is predominantly expressed in the scattering curve. Although the oscillatory rheological results in Figure 2 clearly indicate that the transition to a gel in these samples occurs at an SG concentration of about 1.75 g/L, it is not surprising that already the sample with 1 g/L SG obviously exhibits two structural correlation length parameters (ξ of about 2.3 nm and the static correlation length Ξ of about 10 nm), since the polymer solutions in vicinity of the sol–gel transition generally already exhibit a considerable viscoelastic character, which is attributable to strong intermolecular interactions and long-lived entanglements.^49,50^ At larger SG concentrations, the system exhibits a weak gel in line with the oscillatory rheological results, so it is expected that the model shown in eq S4 should give a good fit to the experimental data, as indeed observed in Figure 3.

Note that our SAXS data are unfortunately somewhat limited in terms of experimental resolution (by the *q*_min_ value of our SAXS instrument), so that only a brief onset of excess scattering represented by the second term in eq 2 is seen in the innermost region of the SAXS curves at low values of *q*. If data were available for even lower values of *q*, the fits would probably provide somewhat more consistent results on the magnitudes of the two correlation lengths. Nonetheless, the values around 2.7 and 17 nm for the dynamic and static correlation length, respectively, clearly indicate two distinct structural features that develop in these samples as they transition from sol to gel, a transition that is clearly indicated by the oscillatory rheology results.

Based on these rheological and SAXS results and the research question on how salts and ILs affect the gelation state or the sol–gel transition process of SG solutions, the samples with 1.75 g/L of SG, which is a concentration close to the sol–gel transition in a pure aqueous sample, were used in further experiments. At this SG concentration, the polymer samples already exhibit pseudoplastic features, which can be modified by adding salts or ILs, as discussed later. Note that in practical applications, electrolyte mixtures of mono- and multivalent ions are often present, and thus, the sol–gel transition point can be shifter depending on the type and concentration of charged species. Such a study in mixed salt solutions can be performed in a future project.

### 3.2 Charge Features of SG

Prior to adding electrolytes to the biopolymer, electrophoretic mobilities were determined in the pH range from 3 to 11 and are shown in Figure 4A. Based on the data measured, one can conclude that the SG possesses a slightly negative charge in aqueous solution, which is constant in the pH regime investigated, as the same mobilities (−3.1 × 10^–9^ m^2^/(V s)) were determined within the experimental error. This is in line with previous reports on the charging properties of SG, which was found to be of nonionic character and thus applicable in a wide range of pH and salinity in industrial processes such as enhanced oil recovery.^22^ Note that line charge density of SG can be increased by oxidation and subsequent formation of carboxylic groups.^7^

**Figure 4:**
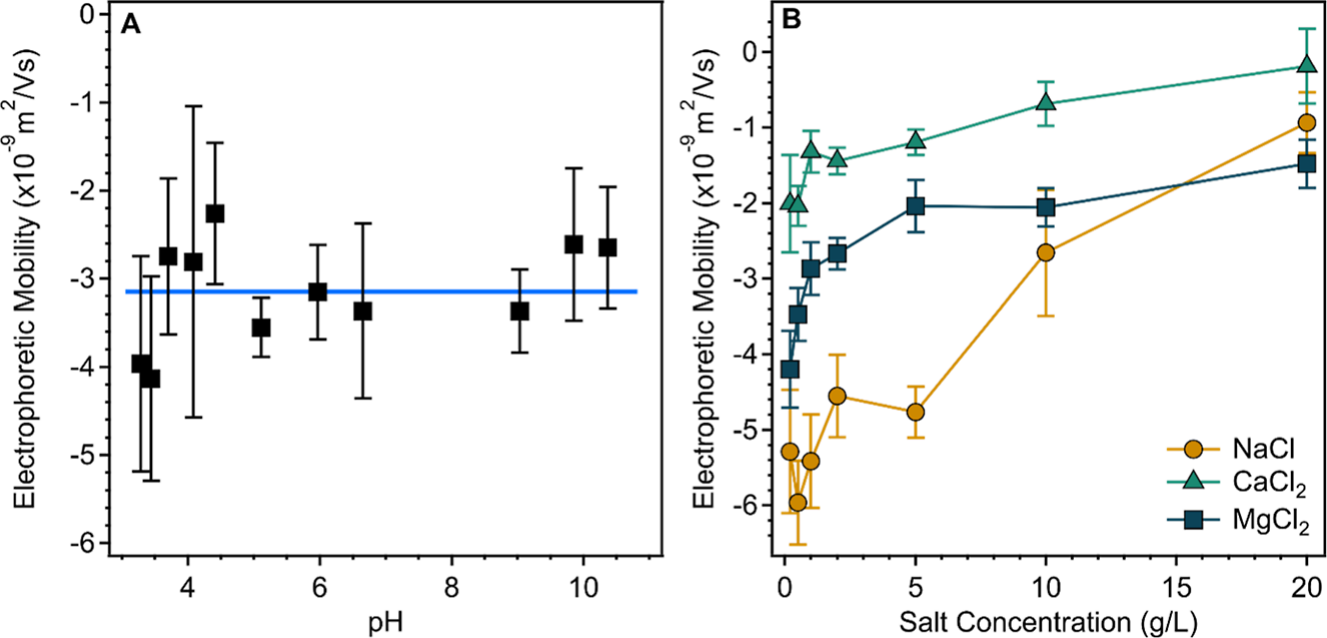
Electrophoretic mobilities of SG solutions at different pH (A) and salt solutions (B). The polymer concentration was 1.75 g/L, and the ionic strength was 1 mM in (A), while the pH was neutral in (B).

Another set of experiments were carried out to assess the influence of mono and divalent salts on the electrophoretic mobility of SG over a wide range of salinity at neutral pH (Figure 4B). In general, the mobilities increased as the salt concentration increased in each case. Such an increase was the most pronounced for NaCl, while in the presence of CaCl_2_ and MgCl_2_, the mobilities were always more positive at the same concentrations. This is in agreement with the stronger charge screening of polymers by dissolved multivalent ions and predicted by the Poisson–Boltzmann theory.^65–67^ In addition, extent of ion condensation into the SG chain^68^ may vary significantly depending on the affinity of the ions to the polymer, and this phenomenon can be responsible for the clear difference between the mobilities in CaCl_2_ and MgCl_2_ solutions at the same salt concentrations. Accordingly, ion specific effects gave rise to the stronger interactions between calcium­(II) ions and SG, leading to less negative charge compared with the magnesium­(II) ions. Similar differences in the specific behavior of inorganic ions in the presence of polymers and surfaces have been experienced and explained with the Hofmeister effect.^69,70^

### 3.3 Rheological and Structural Features of SG in Salt Solutions

To explore the flow properties in the presence of salts ions, the samples with 1.75 g/L SG (originally appearing as gels in pure water) were prepared with NaCl (Figure 5A), CaCl_2_ (Figure 5B), or MgCl_2_ (Figure 5C) electrolytes in the concentration range of 0.2–200 g/L. Since good agreement in the tendencies of data obtained in rotational and oscillatory mode in aqueous SG solutions without added salts, only oscillatory rheological measurements were performed in this case.

**Figure 5:**
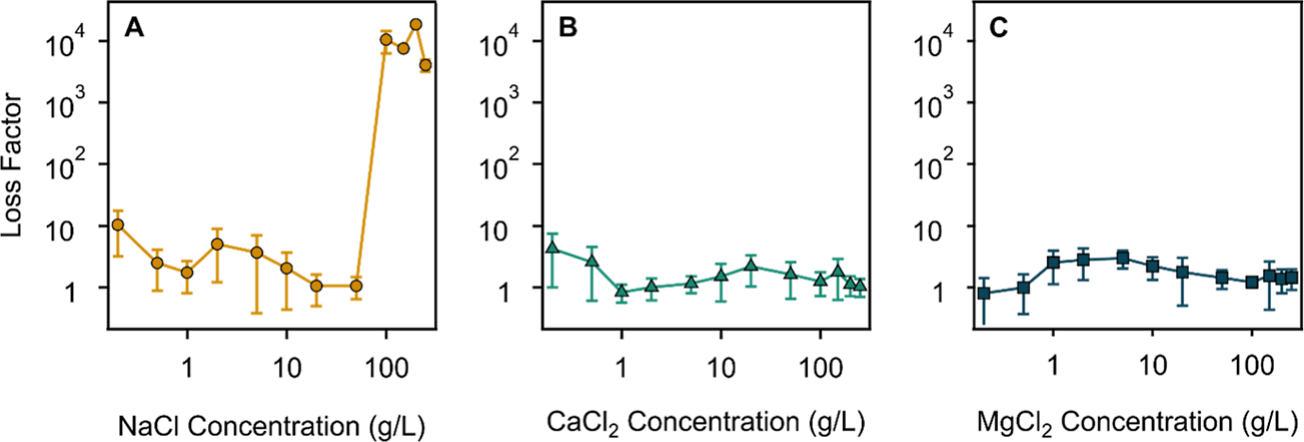
Loss factor data for SG in NaCl (A), CaCl_2_ (B), and MgCl_2_ (C) solutions. The measurements were performed at 1.75 g/L SG concentration. The lines are eye guides. The corresponding storage and loss moduli values are shown in Table S3 in the Supporting Information.

In general, the loss factor values are slightly higher at low salt concentrations in all studied samples in comparison to the one determined for SG in pure water (Figure 2). However, such a difference is not significant and clearly shows that these samples still exhibit a strong elastic character at low salinity for all electrolytes even though their viscous character seem to prevail. For NaCl solutions, the data indicate a considerable elastic character of polymer structures in this system existing in the range between 0.2 g/L and 50 g/L salt concentration. However, the values of loss factor increased steeply at NaCl concentrations above 50 g/L and stabilized at very high values at salt levels beyond 100 g/L, indicating a strong increase of viscous character of these solutions and considerable loss of the SG structure coherency in this high concentration regime of NaCl. Such a feature seems to be significantly higher in this case as was observed and reported similarly for synthetic polymers studied in the same way.^18^ At this elevated NaCl concentration, the SG system is obviously a solution, which nicely conforms to the fact that the SAXS scattering curve of this system with 100 g/L NaCl shown in Figure 6A could be fitted perfectly by eq S3, which is typically able to describe the structural features of diluted polymer solutions with weak intermolecular interactions. The obtained value of the dynamic correlation length of SG in this system was found to be 2.6 nm and is given in Table 2 together with the values of the fitting parameters obtained for other SG samples containing different additives.

**Figure 6:**
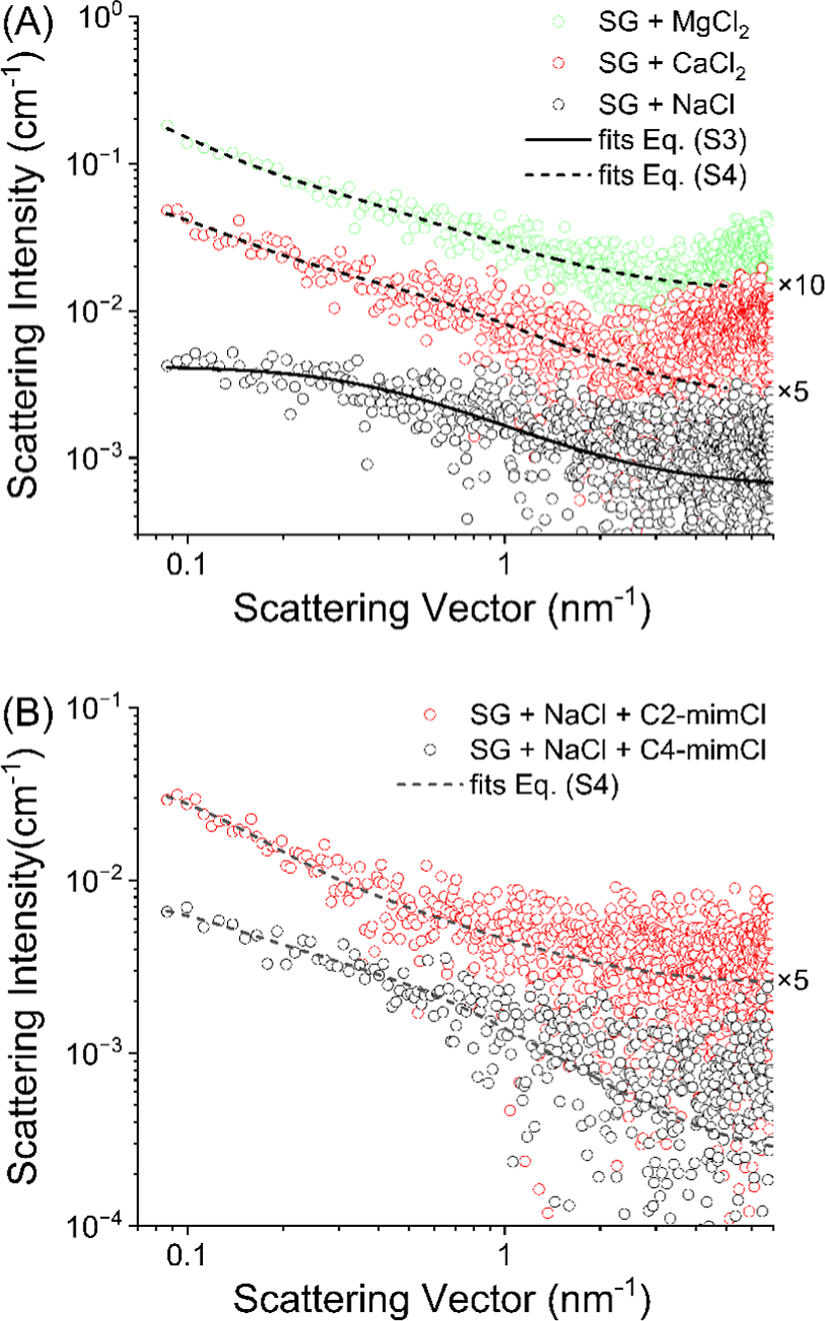
Experimental SAXS curves of aqueous samples with 1.75 g/L of SG and 100 g/L of electrolyte: (A) NaCl, CaCl_2_, and MgCl_2_ (symbols) and (B) NaCl with additional 30 mmol/L of ILs C2-mimCl and C4-mimCl, with the corresponding fits according to eq S3 (solid line) and according to eq S4 (dashed lines). For the sake of clarity, some SAXS curves are shifted upward by a specified factor. Error bars in the low *q* regime are within the size of the symbols.

**Table 2:** Resulting Fitting Parameters of SAXS Data for Samples with 1.75 g/L of SG and 100 g/L of Simple Electrolyte and 30 mmol/L of IL C2-mimCl and C4-mimCl: the Dynamic Correlation Length, ξ, and the Static Correlation Length, Ξ

sample	ξ (nm)	Ξ (nm)
SG + NaCl	2.6 ± 0.3	
SG + CaCl_2_	2.2 ± 0.4	9 ± 2
SG + MgCl_2_	3.6 ± 0.1	13 ± 4
SG + NaCl + C2-mimCl	2.2 ± 0.5	7 ± 3
SG + NaCl + C4-mimCl	2.3 ± 0.9	7 ± 1

Furthermore, the fits to the SAXS data for the SG solutions containing divalent electrolytes are also shown in Figure 6A and reveal that the use of eq S4 containing the two correlation lengths, i.e., dynamic ξ and static Ξ, typical for the coherent elastic or gel-like polymer structure was necessary to fit these data appropriately. The resulting values of the parameters in Table 2 indicate structural features similar to those found already in the SG system in pure water. Note that also these results are somewhat hindered by the limited SAXS experimental resolution and by generally low scattering power of these studied systems. Nevertheless, SAXS parameters determined clearly show the discussed characteristic differences between the studied samples and are also in very good agreement with the oscillatory rheological results obtained for these samples with divalent cations presented in the form of loss factor data in Figure 5B,C. Namely, these loss factor values remained constant within the experimental error in the overall salt concentration regime studied and indicated a considerable elastic character of SG structures in these samples containing ions of CaCl_2_ and MgCl_2_. This result somehow contrasts with the findings for the NaCl system and indicates that the divalent ions stabilize the strong elastic character of these solutions even at high salinity. Such a stabilization may occur through the ion pair formation,^71^ complexation,^72^ or condensation;^68^ nevertheless, one cannot unambiguously claim the underlining mechanism based solely on the presented results. These findings imply that SG solutions maintain some characteristics very similar to elastic properties of a gel-like structure in the presence of divalent metal ions even at elevated concentrations, which is of practical importance in application, wherever electrolyte mixtures are used.

It is evident from the above data that sodium­(I) ions possess a stronger ability to break the coherent SG structure at high salt concentrations compared to the divalent ions. This behavior can be explained as follows. First, the polymer exhibits a high degree of hydrophilicity, and the presence of functional groups facilitates electrostatic screening interactions. This feature may enhance the solubility and stability of individual SG chains in NaCl solutions at higher ionic strengths. In contrast, divalent cations typically induce bridging effects between polymer chains leading to more coherent and interlinked structures owing to ion specific effects through the development of coordination bonds, for instance. As a consequence, the viscosity increases in the presence of divalent metal ions, unlike in monovalent salt solutions.^73^ Second, individual SG chain formation is facilitated by inert electrolytes such as NaCl, as the ionic strength influences inter- and intramolecular interactions differently than that for divalent cations interacting specifically with the functional groups. Thereby, NaCl can maintain solution (and not gel-like) properties under high salt concentrations. Third, the mechanical properties of SG can be improved when interacting with NaCl, which may be attributed to the ability to alter the entropic and enthalpic contributions to the system. In direct comparisons, the mechanical strength of SG in hydrogels has been reported to be enhanced significantly by interactions with monovalent cations, underscoring the notable difference in behavior in the presence of NaCl versus CaCl_2_ and MgCl_2_.^74^ While the concentration of monovalent salts is typically higher than for divalent metal ions, an already smaller amount of the latter ones may lead to the formation of coherent gel-like structures desired in many applications.

Recent studies^75,76^ revealed that divalent ions have a relatively minor impact on the minimum SG concentration required for the formation of a stable gel network. These results imply that the presence of divalent metal ions may not remarkably shift the sol–gel transition concentration compared with pure monovalent salts. Note that, however, the precise polymer concentration corresponding to the sol–gel transition must be determined in each individual system, considering the salt concentration relevant to the specific application. Due to the complexity and variability of formulation conditions in industrial processes, application-specific measurements should be performed to optimize performance.

### 3.4 Effect of ILs

As novel additives, ILs were reported to alter rheology features of polymer solutions;^27,42,77^ however, their interaction with SG was not studied so far. First, oscillation rheology measurements of SG gels were carried out with two ILs differing in the alkyl chain length (Scheme 1) in the concentration range of 0.3–30 mM. The results are shown in the form of loss factor data in Figure 7.

**Figure 7:**
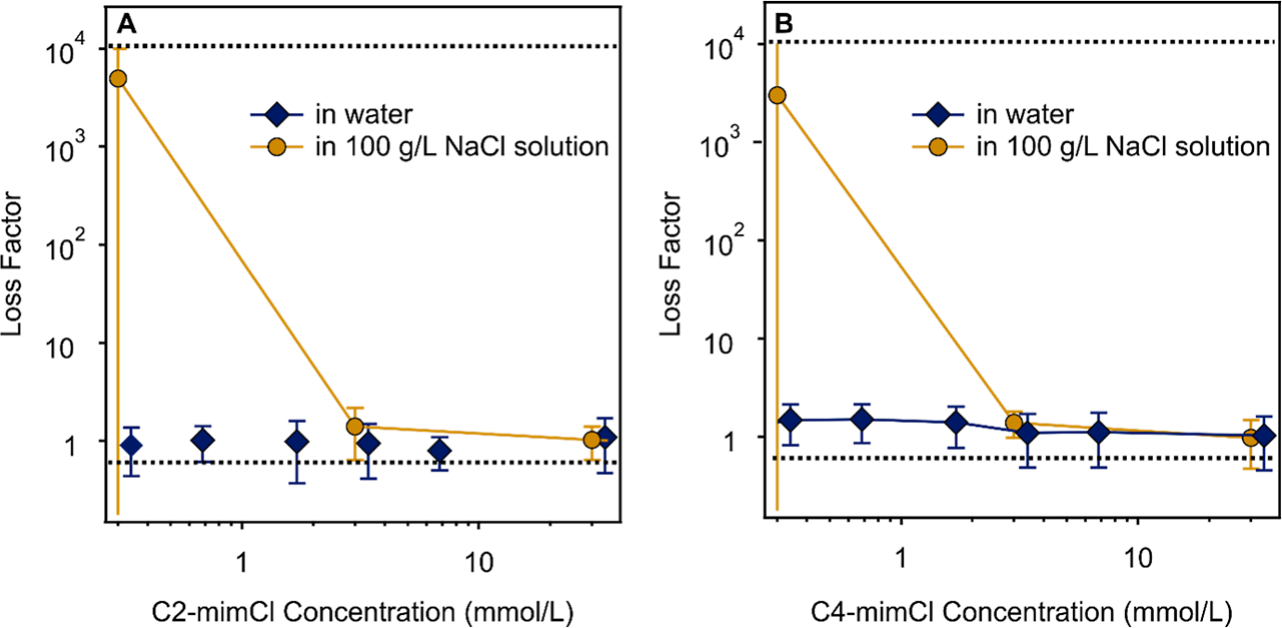
Oscillation rheology-based loss factor data of SG solutions (1.75 g/L polymer concentration) in the presence of C2-mimCl (A) and C4-mimCl (B) in water and with 100 g/L NaCl as added salt. The solid lines guide the eyes, and the bottom dashed line corresponds to the loss factor value of the SG solution without any added chemical agents, while the upper ones to the SG in 100 g/L NaCl solution. The corresponding storage and loss moduli values are given in Tables S4A and S5B.

The presence of ILs does not seem to change the coherence of the structure of the SG in these samples, as indicated by the loss factor data close to unity within experimental error. This observation is very similar to the one made in the case of the divalent ions above and assumes advantageous molecular interactions between the ILs and the SG chains. Note that the difference in the alkyl chain length did not cause noticeable deviations, i.e., the same tendencies were measured in the loss factor data with both C2-mimCl and C4-mimCl. Accordingly, the interaction between the macromolecules is maintained, even in the presence of ILs. Also these results are strongly supported by the experimental SAXS data shown in Figure 6B and Table 2, where one can also see the results of the corresponding fits according to eq S4, proving the considerable elastic character of the SG structure in these samples with moderate concentrations of ILs.

As shown before in Figure 5A, SG loses a strong portion of its elastic character at higher NaCl concentrations, and at 100 g/L, the SG forms an incoherent sol. Adding and increasing the concentration of either C2-mimCl (Figure 7A) or C4-mimCl (Figure 7B) in SG solution with 100 g/L NaCl significantly changes the oscillatory rheological features. At 3 mM IL concentration, the sol state gains a considerable elastic character—the loss factor approaches unity above this IL concentration value. In other words, the gel-like structure of SG, which disappeared upon addition of 100 g/L NaCl reformed in the presence of the ILs studied. It is likely that the ILs form hydrogen bonds with the SG chains, similar to other IL-polysaccharide systems reported earlier,^78^ and such a hydrogen bonding network stabilizes the coherent structure of SG even in the presence of 100 g/L NaCl. Such a phenomenon underlines that ILs at appropriate concentrations can alter the rheological tendencies in SG solutions, even in the presence of additives, which stabilize the sol state of the polymer.

## 4 Conclusions

In conclusion, the rheological and charge features of SG solutions can be altered with inorganic salts and ILs. Results of rotational and oscillatory rheological measurements indicated that the sol–gel transition of the biopolymer SG in aqueous solutions was detected in the concentration range 1.50–1.75 g/L in the absence of any additives. The SG possesses limited charge and can be considered a nonionic polymer in a wide pH range, while addition of divalent metal ions can even lower such a line charge density, as revealed by electrophoretic studies. Divalent metal ions such as calcium­(II) and magnesium­(II) as well as imidazolium-based ILs stabilized the exhibited strong elastic character of the pseudoplastic solutions of SG in a broad concentration regime studied. The coherent structure of SG is also maintained up to a high concentration of NaCl, but it collapses in the range above 50 g/L of NaCl. However, the presence of ILs in concentrations above 3 mM led to the reformation of elastic coherent structure of SG even in the presence of 100 g/L NaCl. These results give important insights into altering the rheological nature of SG solutions by additives such as inorganic salts or ILs. The results are of particular importance in applications in which SG solutions of high viscosity are desired, for instance, in enhanced oil recovery or food thickening procedures.

## Supplementary Material



## Notes

Rheology and SAXS data and fitting protocols for the latter method (PDF)
