# Supplementary material for: Structure of the Scleroglucan Biopolymer in Aqueous Solutions of Inorganic Salts and Ionic Liquids
Source: ACS Omega. 2025 Jun 11;10(24):25884–93. doi: 10.1021/acsomega.5c02212 (PMC12199037; doi:10.1021/acsomega.5c02212)
Supplement: Supplementary file 1 [file ao5c02212_si_001.pdf]

Supporting Information (SI)

Structure of Scleroglucan Biopolymer in Aqueous Solutions of Inorganic Salts and Ionic Liquids

Zsófia Vargáné Árok,[‡] *Gábor Dávid Vass,*[‡] *Andrej Jamnik,*[§] *Matija Tomšič,*[§] *and Istvan Szilagyi**[,‡]

[‡]MTA-SZTE Momentum Biocolloids Research Group, Department of Physical Chemistry and Materials Science, Interdisciplinary Research Center, University of Szeged, H-6720 Szeged, Hungary

[§]Faculty of Chemistry and Chemical Technology, University of Ljubljana, Večna pot 113, SI-1000 Ljubljana, Slovenia

*Corresponding Author. E-mail: szistvan@chem.u-szeged.hu

Fitting Expressions for Experimentally Smeared Small-Angle X-ray Scattering Data. The obtained SAXS data were experimentally smeared due to the finite dimensions of the primary X-ray beam in the SAXS instrument utilizing the block-collimation system. The experimental smearing effects can be properly handled during the SAXS data fitting procedure, if only the theoretical scattering intensity expression (e.g. Eq. (1) and Eq. (2) from the paper) is previously properly modified to consider the experimental smearing effects and the experimental intensity profile of the primary X-ray beam is known.

The smearing due to the finite length of the primary beam is described by the following equation:[1]

$$\tilde{I}_{\mathrm{v}}(m)=\int_{-\infty}^{\infty} P(t)\, I\left(\sqrt{m^2+t^2}\right) dt = 2\int_{0}^{\infty} P(t)\, I\left(\sqrt{m^2+t^2}\right) dt\,, \tag{S1}$$

where $\tilde{I}_{\mathrm{v}}(m)$ represents the experimentally smeared scattering intensity at scattering vector m in the direction along the detector, $P(t)$ the profile of the X-ray primary beam as a function of the sacttering vector t in a direction perpendicular to the direction of m, and $I\left(\sqrt{m^2+t^2}\right)$ the theoretical scattering function, which is in our case given by the expressions Eq. (1) and Eq. (2) in the paper. The primary beam profile, $P(t)$, was measured during the alignment procedure of our SAXS instrument and is shown in Figure S1, where it is fitted and approximated by two lines yielding the following analytical $P(t)$ function for the profile of the primary beam:

$$P(t)=\begin{cases} 0\le t<t_1, & \dfrac{(P_1-P_0)}{(P_0t_1+P_1t_2)\cdot t_1}\cdot t+\dfrac{P_0}{P_0t_1+P_1t_2} \\ t_1\le t<t_2, & \dfrac{P_1\cdot(t_2-t)}{(P_0t_1+P_1t_2)\cdot(t_2-t_1)} \\ t_2\le t, & 0 \end{cases}\,, \tag{S2}$$

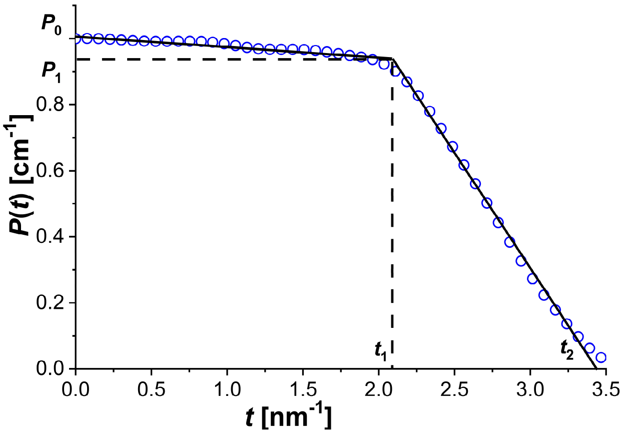

Figure S1. The experimental $P(t)$ length profile of the X-ray primary beam used in this study (circles) and approximated lines (solid line) with the notation of the parameters used in Eq. (S2).

where P_0 and P_1 equal the intensity of the profile at $t=0$ and $t=t_1$, respectively, with t_1 representing the value of the scattering vector t, where the two approximated lines intersect. Similarly, t_2 corresponds to the value of t where the length profile adopts the value of zero. The values of the parameters that we obtained for the profile of the primary beam in our study were the following: P_0 = 1.0055 cm^{-1}, P_1 = 0.94812 cm^{-1}, t_1 = 2.0833 nm^{-1}, and t_2 = 3.4276 nm^{-1}.

If we substitute Eq. (1) and Eq. (S2) in Eq. (S1), the integrals can be solved analytically and lead to the following expression that can be fitted directly to the experimental SAXS data of the polymer solutions on an absolute scale:

$$\tilde{I}(q)=\frac{C}{(t_2-t_1)(P_0t_1+P_1t_2)}\cdot\left[\frac{1}{2\xi^2}\cdot\left((P_1-P_0)\cdot\left(\frac{t_2}{t_1}-1\right)\cdot\ln\left(\frac{1+(q^2+t_1^2)\xi^2}{1+q^2\xi^2}\right)+P_1\cdot\ln\left(\frac{1+(q^2+t_1^2)\xi^2}{1+(q^2+t_2^2)\xi^2}\right)\right)+\right.$$
$$+\frac{1}{\xi\sqrt{1+q^2\xi^2}}\left(((P_0-P_1)\cdot t_2-P_0t_1)\cdot\arctan\left(\frac{t_1\xi}{\sqrt{1+q^2\xi^2}}\right)+\right.$$
$$\left.\left.+P_1t_2\cdot\arctan\left(\frac{t_2\xi}{\sqrt{1+q^2\xi^2}}\right)\right)\right] \qquad \text{(S3)}$$

Similarly, by applying Eq. (2) and Eq. (S2) to Eq. (S1) and solving the integrals analytically, we obtain the following expression for fitting the experimental SAXS data of the polymer gels on an absolute scale:

$$\tilde{I}(q)=\frac{1}{2(t_2-t_1)(P_0t_1+P_1t_2)}\cdot\left[\frac{A\,\Xi^2}{(1+q^2\Xi^2)^{3/2}}\cdot\left(P_1t_2\cdot\arctan\left(\frac{t_2\Xi}{\sqrt{1+q^2\Xi^2}}\right)+((P_0-P_1)t_2-P_0t_1)\cdot\arctan\left(\frac{t_1\Xi}{\sqrt{1+q^2\Xi^2}}\right)\right)+\right.$$
$$+\frac{2B}{\xi\sqrt{1+q^2\xi^2}}\left(P_1t_2\cdot\arctan\left(\frac{t_2\xi}{\sqrt{1+q^2\xi^2}}\right)+((P_0-P_1)t_2-P_0t_1)\cdot\arctan\left(\frac{t_1\xi}{\sqrt{1+q^2\xi^2}}\right)\right)-$$
$$\left.-\frac{B(P_0-P_1)(t_2-t_1)}{t_1\xi}\ln\left(1+\frac{t_1^2\xi^2}{1+q^2\xi^2}\right)-\frac{P_1B}{\xi^2}\ln\left(\frac{1+(q^2+t_2^2)\xi^2}{1+(q^2+t_1^2)\xi^2}\right)\right] \qquad \text{(S4)}$$

Fits of SAXS Data. In Figure S2 the fits of Eq. (S3) to the SAXS data of pure aqueous SG samples are shown. With the exception of the data for 0.5 g/L of SG in the sample, which was found to behave as a sol, the fits are not considered to be good enough. Therefore, we only show them for the sake of comparison with the fits according to Eq. (S4), which are shown in Figure 3 in the paper and appear to be much better, as they consider the presence of two characteristic correlation lengths in the samples, as is usually observed in case of polymer gels or even in polymer solutions with moderate viscoelastic character.[2,3]

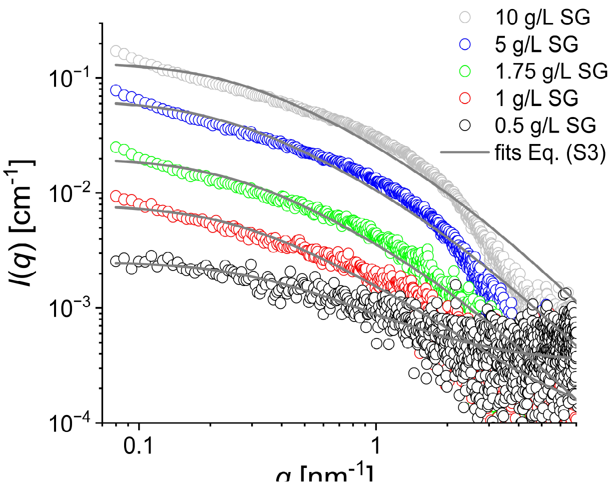

Figure S2. The experimental SAXS curves of pure aqueous samples of SG (symbols) and the corresponding fits according to the Eq. (S3) for polymer solutions.

References:

(1) Glatter, O. Data Treatment. In *Small Angle X-Ray Scattering*, Glatter, O.; Kratky, O., Eds.; Academic Press Inc. London Ltd.: London, **1983**; pp 119–165.

(2) Benigar, E.; Dogsa, I.; Stopar, D.; Jamnik, A.; Kralj Cigić, I.; Tomšič, M., Structure and Dynamics of a Polysaccharide Matrix: Aqueous Solutions of Bacterial Levan. *Langmuir* **2014,** *30* (14), 4172-4182.

(3) Cerar, J.; Jamnik, A.; Tomsic, M., Testing Classical Approach to Polymer Solutions on SAXS Data of lambda-Carrageenan, kappa-Carrageenan and Methylcellulose Systems. *Acta. Chim. Slov.* **2015,** *62* (3), 498-508.

Table S1. The experimental data of flow curves. 1.0 g/L SG in pure aqueous sample was measured between 1 and 1000 1/s shear rate (SR) three times (M1, M2, M3) to calculate standard deviation (SD) and relative standard deviation (RSD) values.

SR (1/s)	M1 (Pa)	M2 (Pa)	M3 (Pa)	Average (Pa)	SD (Pa)	RSD
1	0.0823	0.0800	0.0873	0.0832	0.0037	4.45
1.44	0.0951	0.0931	0.1002	0.0961	0.0036	3.79
2.07	0.1095	0.1080	0.1146	0.1107	0.0035	3.13
2.98	0.1253	0.1236	0.1303	0.1264	0.0035	2.73
4.28	0.1439	0.1426	0.1482	0.1449	0.0029	2.02
6.16	0.1650	0.1633	0.1692	0.1658	0.0030	1.83
8.86	0.1891	0.1884	0.1935	0.1903	0.0028	1.45
12.7	0.2189	0.2176	0.2229	0.2198	0.0028	1.27
18.3	0.2535	0.2530	0.2580	0.2549	0.0028	1.09
26.4	0.2962	0.2963	0.3003	0.2976	0.0023	0.78
37.9	0.3487	0.3496	0.3527	0.3503	0.0021	0.59
54.6	0.4149	0.4162	0.4188	0.4166	0.0020	0.48
78.5	0.4993	0.5006	0.5026	0.5008	0.0017	0.34
113	0.6082	0.6091	0.6111	0.6094	0.0015	0.25
162	0.7486	0.7499	0.7508	0.7498	0.0011	0.14
234	0.9333	0.9321	0.9351	0.9335	0.0015	0.16
336	1.1758	1.1732	1.1759	1.1750	0.0015	0.13
483	1.4976	1.4935	1.4962	1.4958	0.0021	0.14
695	1.9305	1.9235	1.9290	1.9277	0.0037	0.19
1000	2.5143	2.5088	2.5181	2.5137	0.0047	0.19

Table S2. The average of measured storage (G') and loss (G") moduli with standard deviation (SD) values for SG solutions at different polymer concentrations. These data were used to calculate the loss factor values in Figure 2.

SG concentration (g/L)	G' (Pa)	SD for G' (Pa)	G" (Pa)	SD for G" (Pa)
0.1	0.0000139	0.0000084	0.278	0.167
0.25	0.0000151	0.0000043	0.302	0.087
0.5	0.0000180	0.0000025	0.360	0.049
1	0.018	0.037	0.349	0.040
1.25	0.221	0.101	0.380	0.022
1.5	0.363	0.175	0.467	0.022
1.75	1.140	0.192	0.662	0.016
2	1.460	0.247	0.770	0.037
2.25	1.920	0.428	0.871	0.064
2.5	2.320	0.390	1.061	0.013
5	8.520	1.523	2.620	0.135

Table S3. The average of measured storage (G’) and loss (G”) moduli with standard deviation (SD) values for SG in NaCl, $CaCl_2$, $MgCl_2$ solutions. The measurements were performed at 1.75 g/L SG concentration. These data were used to calculate loss factor values shown in Figure 5.

**Salt concentration (g/L)**	**G’ in NaCl (Pa)**	**SD for G’ in NaCl (Pa)**	**G” in NaCl (Pa)**	**SD for G” in NaCl (Pa)**	**G’ in $CaCl_2$ (Pa)**	**SD for G’ in $CaCl_2$ (Pa)**	**G” in $CaCl_2$ (Pa)**	**SD for G” in $CaCl_2$ (Pa)**	**G’ in $MgCl_2$ (Pa)**	**SD for G’ in $MgCl_2$ (Pa)**	**G” in $MgCl_2$ (Pa)**	**SD for G” in $MgCl_2$ (Pa)**
0.2	0.292	0.173	0.404	0.021	0.266	0.134	0.467	0.023	0.506	0.373	0.417	0.031
0.5	0.212	0.097	0.394	0.022	0.326	0.141	0.506	0.021	0.405	0.227	0.400	0.061
1	0.290	0.134	0.390	0.018	0.655	0.186	0.498	0.015	0.335	0.181	0.443	0.026
2	0.144	0.079	0.394	0.011	0.549	0.152	0.495	0.029	0.256	0.114	0.425	0.018
5	0.143	0.078	0.258	0.044	0.389	0.143	0.461	0.021	0.261	0.143	0.432	0.040
10	0.297	0.203	0.355	0.021	0.407	0.215	0.447	0.023	0.323	0.143	0.450	0.035
20	0.429	0.364	0.281	0.022	0.393	0.236	0.530	0.023	0.377	0.161	0.520	0.016
50	0.386	0.155	0.347	0.025	0.486	0.215	0.562	0.025	0.721	0.273	0.605	0.030
100	0.0000202	0.0000047	0.400	0.054	0.589	0.154	0.660	0.020	0.822	0.165	0.703	0.013
150	0.0000361	0.0000005	0.488	0.012	0.399	0.137	0.578	0.020	0.565	0.230	0.669	0.019
200	0.0000197	0.0000024	0.390	0.048	0.612	0.159	0.624	0.018	0.620	0.178	0.749	0.170
250	0.0000618	0.0000023	0.406	0.064	0.711	0.193	0.665	0.062	0.637	0.179	0.857	0.047

Table S4. The average of measured storage (G') and loss (G") moduli with standard deviation (SD) values for SG solutions (1.75 g/L polymer concentration) in the presence of C2-mimCl and C4-mimCl in water. The data correspond to the loss factor values in Figure 7A.

IL concentration (mmol/L)	G' of SG and C2-mim in water (Pa)	SD for G' of SG and C2-mim in water (Pa)	G" of SG and C2-mim in water (Pa)	SD for G" of SG and C2-mim in water (Pa)	G' of SG and C4-mim in water (Pa)	SD for G' of SG and C4-mim in water (Pa)	G" of SG and C4-mim in water (Pa)	SD for G" of SG and C4-mim in water (Pa)
0.34	0.875	0.230	0.706	0.091	0.616	0.216	0.648	0.022
0.68	0.717	0.218	0.647	0.039	0.824	0.531	0.636	0.024
1.71	0.784	0.282	0.639	0.047	0.799	0.505	0.628	0.025
3.41	0.758	0.219	0.622	0.026	0.650	0.233	0.606	0.071
6.82	0.873	0.219	0.637	0.040	0.717	0.261	0.628	0.022
34.10	0.626	0.175	0.593	0.018	0.608	0.148	0.572	0.027

Table S5. The average of measured storage (G') and loss (G") moduli with standard deviation (SD) values for SG solutions (1.75 g/L polymer concentration) in the presence of C2-mimCl and C4-mimCl in water with 100 g/L NaCl as added salt. The loss factor data in Figure 7B were calculated from these moduli values.

IL concentration (mmol/L)	G' of SG and C2-mim in 100 g/L NaCl solution (Pa)	SD for G' of SG and C2-mim in 100 g/L NaCl solution (Pa)	G" of SG and C2-mim in 100 g/L NaCl solution (Pa)	SD for G" of SG and C2-mim in 100 g/L NaCl solution (Pa)	G' of SG and C4-mim in 100 g/L NaCl solution (Pa)	SD for G' of SG and C4-mim in 100 g/L NaCl solution (Pa)	G" of SG and C4-mim in 100 g/L NaCl solution (Pa)	SD for G" of SG and C4-mim in 100 g/L NaCl solution (Pa)
0.3	0.362	0.238	0.491	0.013	0.184	0.110	0.444	0.019
3	0.399	0.143	0.472	0.079	0.592	0.192	0.507	0.067
30	0.528	0.134	0.498	0.017	0.455	0.177	0.499	0.021
